# Reevaluation of the Effect of Iodine on Thyroid Cell Survival and Function Using PCCL3 and Nthy-ori 3-1 Cells

**DOI:** 10.1210/jendso/bvaa146

**Published:** 2020-09-28

**Authors:** Tomomi Kurashige, Mika Shimamura, Yuji Nagayama

**Affiliations:** Department of Molecular Medicine, Atomic Bomb Disease Institute, Nagasaki University, Nagasaki, Japan

**Keywords:** thyroid, iodine, autophagy, NIS, TSHR

## Abstract

The appropriate amount of iodine is critical for normal function of thyroid cells synthesizing thyroid hormones. Although normal thyroid cell lines such as rat PCCL3 and FRTL5 and human Nthy-ori 3-1 have been widely used for in vitro studies on physiological and pathophysiological effects of iodine on thyroid cells, we have recently pointed out the critical differences between FRTL5/PCCL3 cells and Nthy-ori 3-1 cells. Therefore, we here directly compared some of the cellular characteristics—iodine uptake, differentiated status, iodine-induced cytotoxicity, and iodine-regulation of autophagy—between PCCL3 and Nthy-ori 3-1 cells. PCCL3 cells express messenger RNAs for thyrotropin receptor and sodium/iodine symporter and incorporate iodine in a thyrotropin-dependent manner, whereas Nthy-ori 3-1 cells do not either. Nevertheless, both cells were comparably resistant to iodine cytotoxicity: Only far excess iodine (5 × 10^–2^ M) killed 20% to 40% cells in 24 hours with perchlorate exhibiting no effect, suggesting this cytotoxic effect is due to extracellular iodine. In contrast, a wide range of iodine (5 × 10^–9^ to 5 × 10^–2^ M) induced autophagy in PCCL3 cells, which was abolished by perchlorate, indicating intracellular iodine-induction of autophagy, but this effect was not observed in Nthy-ori 3-1 cells. In conclusion, it is critical to discriminate the effect of iodine incorporated into cells from that of extracellular iodine on thyroid cells. Iodine-uptake competent thyroid cells such as PCCL3 and FRTL5 cells, not Nthy-ori 3-1 cells, should be used for studies on iodine effect on thyroid cells.

The thyroid follicular epithelial cells actively incorporate iodine from the bloodstream to synthesize thyroid hormones, but excessive and deficient iodine both cause thyroid dysfunction. Normal thyroid cell lines are very useful to study in vitro physiological and pathophysiological effects of iodine on thyroid cell survival and function. They include rat normal thyroid cell lines, PCCL3 [1] and FRTL5 [2], and a human cell line, Nthy-ori 3-1 [3]. Nthy-ori 3-1 cells have recently been used to show iodine-inductions of cell death [4-6] and of endoplasmic reticulum stress [7], and inhibition of autophagy [8]. However, we have recently pointed out the critical differences between FRTL5/PCCL3 cells and Nthy-ori 3-1 cells and expressed our concern that the latter may not be suitable for studies on in vitro effect of iodine on thyroid cells [9]. For example, we and others have previously shown that the former take up iodine [10, 11], whereas the latter do not [12, 13]. Therefore, we here directly compared some cellular characteristics such as the ability to take up iodine, differentiated status, the sensitivity to iodine-induced cytotoxicity, and iodine-regulation of autophagy between PCCL3 and Nthy-ori 3-1 cells.

## 1. Materials and Methods

### A. Cell Line Used

**Figure 1.**
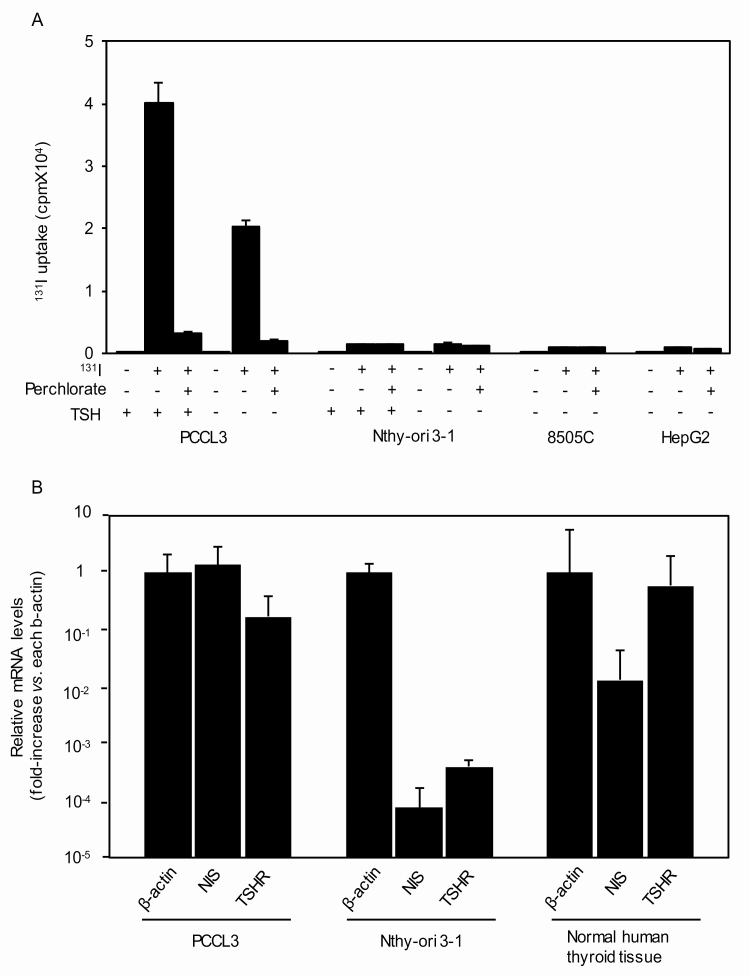
^131^Iodine (^131^I) uptake in PCCL3, Nthy-ori 3-1, 8505C, and HepG2 cells, and thyrotropin receptor (TSHR) and sodium/iodine symporter (NIS) messenger RNA (mRNA) expression in PCCL3 and Nthy-ori 3-1 cells. A, Cells were cultured in the presence or absence of thyrotropin (TSH) and/or perchlorate and then incubated with 10 μCi/mL ^131^I for 30 minutes. ^131^I taken up into the cells was measured as described in “Materials and Methods.” Data are means ± SD (n = 3). B, Total RNA was extracted from PCCL3 and Nthy-ori 3-1 cells and subjected to RT-PCR for TSHR, NIS, and β-actin mRNAs as described in “Materials and Methods.” Data are means ± ranges (n = 2).

A rat normal thyroid cell line PCCL3 [1] was previously described [14] and maintained in Coon’s modified F-12 medium supplemented with 5% fetal bovine serum (FBS), antibiotics and 3H (2 U/L bovine thyrotropin [TSH], 5 U/L insulin, and 5 mg/L transferrin) or 2H (insulin and transferrin) (all from Sigma-Aldrich). A human normal cell line Nthy-ori 3-1 [3], obtained from Health Protection Agency Culture Collections, was cultured in RPMI (Roswell Park Memorial Institute) medium 1640 with 10% FBS and antibiotics (and 2 U/L TSH in Fig. 1). The former was spontaneously immortalized and its growth is totally TSH dependent, whereas the latter is derived from an HTori-3 cell line that was immortalized by transfection of simian virus 40 T-antigen and can grow without TSH [9]. Human anaplastic thyroid cancer cell line 8505C and human hepatocellular cancer cell line HepG2 were obtained from RIKEN Bioresource Center, cultured in RPMI 1640 with 10% FBS and antibiotics, and used as controls.

### B. ^131^Iodine Uptake

PCCL3 cells were maintained in the presence of 3H or incubated with 2H for 3 days, and Nthy-ori 3-1 cells were maintained in the regular medium or incubated with 2 U/L TSH for 3 days. A total of 5 × 10^4^ cells in a 96-well plate were incubated with 10 μCi/mL (37 kBq/μCi) ^131^iodine (^131^I; PerkinElmer) for 30 minutes. Following extensive washing with phosphate buffered saline and resuspension by trypsinization, ^131^I incorporated into the cells was measured using the automatic gamma counter 2470 WIZARD2 (PerkinElmer). Perchlorate (NaClO_4_) was added to the culture medium at the final concentration of 5 μM [15] 1 hour before ^131^I addition to block ^131^I uptake.

### C. Quantitative Real-Time Polymerase Chain Reaction for Thyrotropin Receptor and Sodium/Iodine Symporter Expression

Total RNA extraction and complementary DNA synthesis were performed with PCCL3 cells maintained with 3H and Nthy-ori 3-1 cells incubated with 2 U/L TSH for 3 days as previously described [16]. Complementary DNA synthesized from total RNA from normal human thyroid tissue in the previous study [16] was also used. Polymerase chain reaction (PCR) was then carried out on a Thermal Cycler Dice Real-time system (Takara). The primer pairs used were previously described; human thyrotropin receptor (TSHR) (length of a PCR product; 213 base pairs [bp]) [17], human sodium/iodine symporter (NIS) (420 bp) [18], rat TSHR (302 bp) [19], rat NIS (529 bp) [20], and β-actin (105 bp) [21]. The PCR condition was 40 cycles of denature at 95°C for 15 seconds, annealing at 55°C for 15 seconds, and an extension at 72°C for 30 seconds. The cycle threshold values, which were determined using a second derivative, were used to calculate the normalized expression of the indicated messenger RNAs (mRNAs) using Q-Gene software using β-actin for normalization. The PCR product sizes were also confirmed with 1.5% agarose gel electrophoresis.

### D. Cell Viability Assay

A total of 1 × 10^4^ cells in a 96-well plate were incubated for 24 to 48 hours and then treated with up to 5 × 10^–2^ M NaI for 24 hours. In PCCL3 cells maintained with 3H, perchlorate was added to the culture medium (final concentration: 5 μM) 1 hour before NaI addition. Cell viability was then measured using a Cell Counting Kit-8 (Dojindo) in accordance with the manufacturer’s protocol.

### E. Monitoring of Autophagy

Autophagic activity was monitored in PCCL3 maintained with 3H and Nthy-ori 3-1 cells maintained in the regular medium by immunofluorescence (IF) and immunoblotting (IB), as previously described [22]. The primary and secondary antibodies for p62, LC3 and anti‒β-actin are also shown in [22-28]. When autophagy is induced, LC3-I is converted to LC3-II by lipidation and recruited to autophagosome from the cytoplasm. Therefore, the amount of LC3-II increases in IB, and punctate staining of LC3 appears in IF. Furthermore, the amounts of p62, a substrate of autophagy, decrease by degradation in the autolysosome, which can be determined by IB and IF (see [22] for more details).

### F. Statistical Analyses

Experiments were repeated twice with essentially same results. Statistical differences between different groups were examined with analysis of variance test and Games-Howell test using SPSS. *P* less than .05 was used to identify statistically significant differences.

## 2. Results

We first confirmed the ability/inability of ^131^I uptake in PCCL3 and Nthy-ori 3-1 cells cultured in the presence or absence of TSH [10, 12, 13]; an anaplastic thyroid cancer cell line 8505C and a hepatocellular carcinoma cell line HepG2 were used as negative controls. We have previously shown that PCCL3 cells incorporated ^131^I in a dose-dependent manner, reaching the peak at 30 minutes in the presence of TSH [10]. Therefore, ^131^I uptake was determined 30 minutes after the addition of ^131^I into the culture medium. As shown in Fig. 1A, PCCL3 cells maintained with 3H clearly incorporated ^131^I, which was completely abolished by 5 μM NaClO_4_ (perchlorate), a competitive inhibitor of NIS, confirming NIS-mediated iodine uptake. Removal of TSH for 3 days reduced ^131^I uptake by approximately 50%, indicating the TSH-dependent action of iodine uptake. On the other hand, Nthy-ori 3-1 cells, irrespective of the presence or absence of TSH, showed almost negligible ^131^I uptake; these low levels of iodine uptake are likely nonspecific because the similarly low levels of ^131^I uptake were also observed in 8505C and HepG2 cells, both of which have been shown not to express NIS [29, 30].

We next examined mRNA expression of thyroid-specific molecules critical for TSH-dependent iodine uptake; namely TSHR and NIS. Thus, mRNAs for TSHR and NIS were readily detectable in PCCL3 cells and human thyroid tissue (a positive control), but extremely low levels of their expression was barely observed in Nthy-ori 3-1 cells (Fig. 1B), supporting the ^131^I uptake results in Fig. 1A.

These results clearly demonstrate the substantial differences in the differentiated status between PCCL3 and Nthy-ori 3-1 cells. PCCL3 cells are well differentiated, expressing TSHR and NIS mRNAs, and able to take up iodine, whereas Nthy-ori 3-1 cells are conversely less differentiated, expressing few mRNAs for these 2 molecules, and unable to take up iodine. Nevertheless, both cell lines have long been used comparably as normal thyroid cell lines.

**Figure 2.**
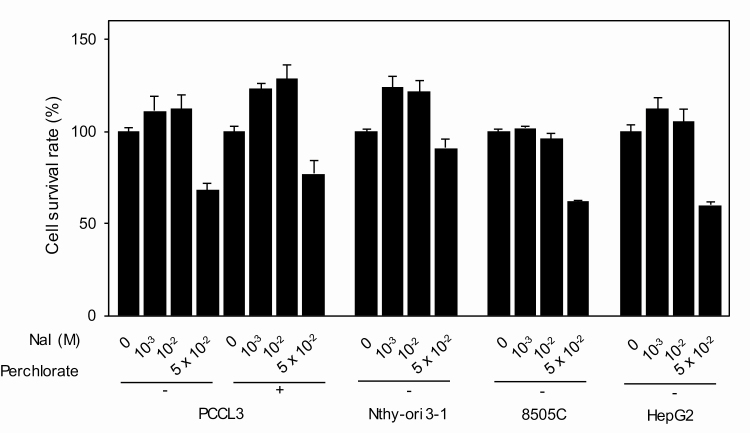
Cytotoxicity of iodine in PCCL3, Nthy-ori 3-1, 8505C, and HepG2 cells. The cells, cultured in the presence or absence of perchlorate, were incubated with up to 5 × 10^–2^ M iodine for 24 hours and cell viability was determined as described in “Materials and Methods.” Data are means ± SD (n = 3).

These results then caused doubts about the previous studies on the effects of iodine on the survival and function of thyroid cells using Nthy-ori 3-1 cells (see “Introduction”) and prompted us to reevaluate and compare some of these results—iodine cytotoxicity and iodine modulation of autophagic activity—using PCCL3 and Nthy-ori 3-1 cells in parallel in our own hands. The concentrations of iodine we used are 5 × 10^–9^ M, the physiological concentration in human sera; 10^–6^ M, the concentration that suppresses iodine uptake in cultured thyroid cells; 10^–3^ M, excess iodine; and 1 and 5 × 10^–2^ M, far excess iodine [10]. Fig. 2 demonstrates the results of the study on iodine cytotoxicity. Up to 10^–2^ M iodine had no effect and only the highest dose of iodine (5 × 10^–2^ M) induced 20% to 40% cell death in 24 hours in both cell lines as well as 8505C and HepG2 cells. Perchlorate had no effect on cell death in PCCL3 cells, indicating that this mild cytotoxic effect of iodine is attributed to extracellular iodine present in the culture medium, not intracellular iodine incorporated into the cells. Thus, both thyroid cell lines as well as 8505C and HepG2 cells are comparably resistant against extracellular iodine cytotoxicity, and iodine incorporated into PCCL3 cells is likely harmless, even when the cells were exposed to far excess iodine (10^–2^ M). To the best of our knowledge, this is the first study on iodine cytotoxicity in PCCL3 cells. Similar results were reported in Nthy-ori 3-1 cells [4-6], human thyroid cells, and FRTL5 cells [31, 32]. Although the mechanism(s) for cytotoxic effect of far excess iodine (5 × 10^–2^ M) in the culture medium is at present unknown, some interaction between iodine and membranous proteins/lipids may explain this effect [33].

**Figure 3.**
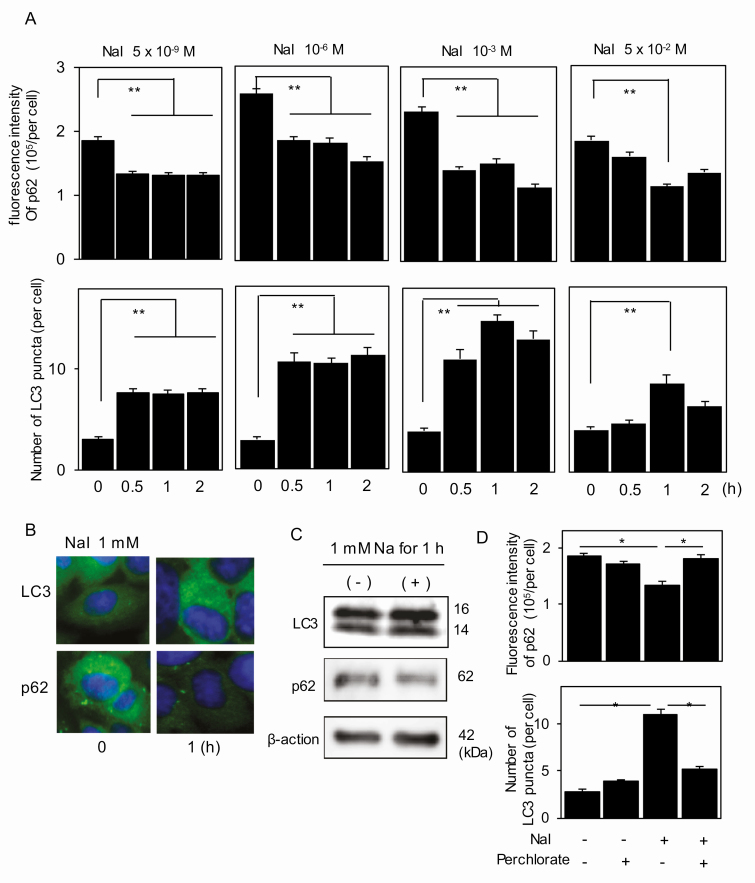
Control of autophagic activity by iodine in PCCL3 cells. A, Cells were incubated with up to 5 × 10^–2^ M iodine for up to 2 hours, and LC3 puncta and P62 fluorescence intensities were quantified by immunofluorescence (IF) as described in “Materials and Methods.” B, Representative photographs for LC3 puncta and p62 fluorescence in the cells incubated with 10^–3^ M iodine for up to 1 hour are shown. C, Western blotting of LC3 and p62 in the cells incubated with 10^–3^ M iodine for 1 hour are shown. D, Cells, cultured in the presence or absence of perchlorate, were incubated with 10^–3^ M iodine for 1 hour, followed by quantification of LC3 puncta and p62 fluorescence intensities by IF as described in “Materials and Methods.” *P less than .05. **P less than .01.

**Figure 4.**
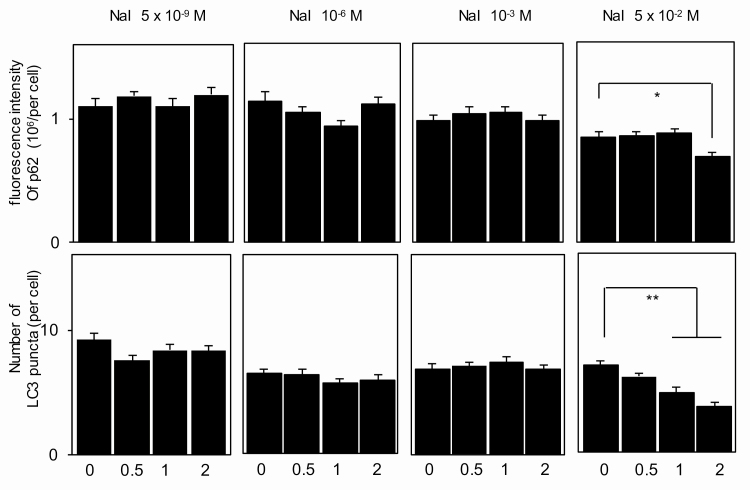
Control of autophagic activity by iodine in Nthy-ori 3-1 cells. Cells were incubated with up to 5 × 10^–2^ M iodine for up to 2 hours, and LC3 puncta and p62 fluorescence intensities were quantified by immunofluorescence as described in “Materials and Methods.” *P less than .05. **P less than .01.

The results of autophagy experiments are shown in Figs. 3 and 4. A wide range of iodine (5 × 10^–9^ M to 10^–3^ M) increased LC3 puncta and deceased p62 fluorescence intensity 0.5 to 2 hours after the addition of iodine in IF in PCCL3 cells (Fig. 3A and 3B), clearly demonstrating induction of autophagy by iodine. The data are also confirmed by IB in cells incubated for 1 hour with 10^–3^ M iodine (Fig. 3C). Perchlorate abolished the effect of iodine (10^–6^ M) on autophagy almost completely, indicating that this effect is exerted by intracellular iodine (Fig. 3D). Far excess iodine (5 × 10^–2^ M) induced the lower degree of autophagy as compared to the lower doses of iodine, which is likely attributed to the cytotoxic effect of far excess iodine as shown in Fig. 2. In contrast, 5 × 10^–9^ M to 10^–3^ M iodine had no effect, and 5 × 10^–2^ M iodine reduced LC3 puncta and increased p62 levels in Nthy-ori 3-1 cells (Fig. 4), again suggesting that far excess iodine suppressed autophagy by its cytotoxicity. Although our data are somewhat different from the previous study, in which 10^–4^ to 5 × 10^–2^ M iodine decreased LC3-II in Western blotting and autophagosome and autolysosome formation with the LC3-RFP-GFP vector in Nthy-ori 3-1 cells [8], our results demonstrate that induction of autophagy by iodine ranging from 5 × 10^–9^ M to 10^–3^ M in PCCL3 cells are due to iodine incorporated into the cells, which cannot be seen in Nthy-ori 3-1 cells that are incapable of incorporating iodine.

The pathophysiological significance of iodine-induced autophagy is unknown. We first thought that iodine increase in reactive oxygen species (ROS) might cause autophagy induction because it is well known that oxidative stress induces autophagy through the PERK-eLF2α-ATF4-CHOP pathway and mitogen-activated protein kinases such as c-Jun N-terminal kinase 1 [34, 35]. Indeed ROS elevation by iodine has previously been reported in FRTL5 and PCCL3 cells; an approximately 2-fold increase in intracellular ROS by 10^–3^ M iodine in 30 minutes and a huge increase in mitochondrial ROS by 10^–2^ M iodine in 2 hours [36] and a biphasic increase (at 2 and 24, not 4, hours) by 10^–4^ M iodine [37]. However, we could not detect ROS increase in iodine-treated PCCL3 cells using 2’,7’- dichlorofluorescin diacetate in our hands (data not shown).

We also found one report inconsistent with our data [38], in which low doses of iodine (10^–8^ to 10^–5^ M) stimulated cell proliferation but a higher dose (10^–3^ M) induced apoptosis and autophagy in the thyroid cancer cell line BCPAP. BCPAP cells were established from a differentiated papillary thyroid cancer, but likely gained an undifferentiated phenotype during long culture [39, 40]. Indeed, BCPAP cells are reported not to express NIS nor take up iodine [12, 39]. Therefore, the previously reported data may indicate a possibility for high sensitivity of BCPAP cells to extracellular iodine.

In conclusion, our results indicate that the in vitro effects of high doses of iodine on thyroid cells so far reported using Nthy-ori 3-1 cells are likely due to iodine present in the culture medium, not iodine incorporated into the cells. Although cultured cell lines are useful tools for in vitro studies on cell behavior and function, appropriate one(s) should be chosen for a certain experiment in general, and in particular, the effect of iodine, especially that incorporated into thyroid cells, should be studied with functional, iodine-uptake competent thyroid cells such as PCCL3 and FRTL5 cells, not Nthy-ori 3-1 cells.

## Data Availability

All data generated during this study are included in this published article.

## Abbreviations

bp: base pair
FBS: fetal bovine serum
IB: immunoblotting
IF: immunofluorescence
LC3: microtubule-associated protein 1 light chain 3
mRNA: messenger RNA
NIS: sodium/iodine symporter
PCR: polymerase chain reaction
ROS: reactive oxygen species
TSH: thyrotropin
TSHR: thyrotropin receptor
U/L: units per liter

## Additional Information

***Disclosure Summary:*** The authors declare no conflict of interest.

## References

[1] CVCL_6712. CVCL_6712 https://web.expasy.org/cellosaurus/CVCL_6712.txt.

[2] CVCL_0265. CVCL_0265 https://web.expasy.org/cellosaurus/CVCL_0265.txt.

[3] CVCL_2659. CVCL_2659 https://web.expasy.org/cellosaurus/CVCL_2659.txt.

[4] Liu H, Zeng Q, Cui Y, et al. The effects and underlying mechanism of excessive iodide on excessive fluoride-induced thyroid cytotoxicity. Environ Toxicol Pharmacol. 2014;38(1):332-340. 25104093 10.1016/j.etap.2014.06.008

[5] Liu H, Zeng Q, Cui Y, et al. The role of the IRE1 pathway in excessive iodide- and/or fluoride-induced apoptosis in Nthy-ori 3-1 cells in vitro. Toxicol Lett. 2014;224(3):341-348. 24231001 10.1016/j.toxlet.2013.11.001

[6] Liu J, Mao C, Dong L, et al. Excessive iodine promotes pyroptosis of thyroid follicular epithelial cells in Hashimoto’s thyroiditis through the rOS-NF-κB-NLRP3 pathway. Front Endocrinol (Lausanne). 2019;10:778. 31824415 10.3389/fendo.2019.00778 PMC6880659

[7] Chen X, Huang H, Liang B, Zhou J Abnormal iodine nutrition-induced ER stress upregulates MCP-1 expression through P38/MAPK signaling pathway in thyroid cells. Biol Trace Elem Res. 2019;191(1):98-103. 30539387 10.1007/s12011-018-1610-9

[8] Xu C, Wu F, Mao C, et al. Excess iodine promotes apoptosis of thyroid follicular epithelial cells by inducing autophagy suppression and is associated with Hashimoto thyroiditis disease. J Autoimmun. 2016;75:50-57. 27448770 10.1016/j.jaut.2016.07.008

[9] Nagayama Y General commentary: excessive iodine promotes pyroptosis of thyroid follicular epithelial cells in Hashimoto’s thyroiditis through the ROS-NF-κB-NLRP3 pathway. Front Endocrinol 2020;11:581. 10.3389/fendo.2020.00581 PMC7484478 32982973

[10] Kurashige T, Shimamura M, Nagayama Y N-Acetyl-L-cysteine protects thyroid cells against DNA damage induced by external and internal irradiation. Radiat Environ Biophys. 2017;56(4):405-412. 28871381 10.1007/s00411-017-0711-8

[11] Grollman EF, Smolar A, Ommaya A, Tombaccini D, Santisteban P Iodine suppression of iodide uptake in FRTL-5 thyroid cells. Endocrinology. 1986;118(6):2477-2482. 3009160 10.1210/endo-118-6-2477

[12] Tuncel M, Aydin D, Yaman E, et al. The comparative effects of gene modulators on thyroid-specific genes and radioiodine uptake. Cancer Biother Radiopharm. 2007;22(3):443-449. 17679169 10.1089/cbr.2006.319.A

[13] Lemoine NR, Mayall ES, Jones T, et al. Characterisation of human thyroid epithelial cells immortalised in vitro by simian virus 40 DNA transfection. Br J Cancer. 1989;60(6):897-903. 2557880 10.1038/bjc.1989.387 PMC2247263

[14] Kurashige T, Shimamura M, Nagayama Y Differences in quantification of DNA double-strand breaks assessed by 53BP1/γH2AX focus formation assays and the comet assay in mammalian cells treated with irradiation and N-acetyl-L-cysteine. J Radiat Res. 2016;57(3):312-317. 26951077 10.1093/jrr/rrw001 PMC4915540

[15] Tran N, Valentín-Blasini L, Blount BC, et al. Thyroid-stimulating hormone increases active transport of perchlorate into thyroid cells. Am J Physiol Endocrinol Metab. 2008;294(4):E802-E806. 18303123 10.1152/ajpendo.00013.2008

[16] Kurashige T, Shimamura M, Yasui K, et al. Studies on expression of aldehyde dehydrogenase in normal and cancerous tissues of thyroids. Horm Metab Res. 2015;47(3):194-199. 25181420 10.1055/s-0034-1387770

[17] Chinnappa P, Taguba L, Arciaga R, et al. Detection of thyrotropin-receptor messenger ribonucleic acid (mRNA) and thyroglobulin mRNA transcripts in peripheral blood of patients with thyroid disease: sensitive and specific markers for thyroid cancer. J Clin Endocrinol Metab. 2004;89(8):3705-3709. 15292293 10.1210/jc.2003-031967

[18] Li W, Ain KB Human sodium-iodide symporter (hNIS) gene expression is inhibited by a trans-active transcriptional repressor, NIS-repressor, containing PARP-1 in thyroid cancer cells. Endocr Relat Cancer. 2010;17(2):383-398. 20228127 10.1677/ERC-09-0156

[19] Endo T, Kobayashi T Thyroid-stimulating hormone receptor in brown adipose tissue is involved in the regulation of thermogenesis. Am J Physiol Endocrinol Metab. 2008;295(2):E514-E518. 18559984 10.1152/ajpendo.90433.2008

[20] Chai W, Yin X, Ren L, et al. Sodium/iodide symporter gene transfection increases radionuclide uptake in human cisplatin-resistant lung cancer cells. Clin Transl Oncol. 2015;17(10):795-802. 26115738 10.1007/s12094-015-1307-x

[21] Knauf JA, Ma X, Smith EP, et al. Targeted expression of BRAFV600E in thyroid cells of transgenic mice results in papillary thyroid cancers that undergo dedifferentiation. Cancer Res. 2005;65(10):4238-4245. 15899815 10.1158/0008-5472.CAN-05-0047

[22] Kurashige T, Nakajima Y, Shimamura M, Yamada M, Nagayama Y Hormonal regulation of autophagy in thyroid PCCL3 cells and the thyroids of male mice. J Endocr Soc. 2020;4(7):bvaa054. 32671315 10.1210/jendso/bvaa054 PMC7347287

[23] RRID:AB_2274121, http://scicrunch.org/resolver/AB_2274121.

[24] RRID:AB_2099233, http://scicrunch.org/resolver/AB_2099233.

[25] RRID:AB_2687531, http://scicrunch.org/resolver/AB_2687531.

[26] RRID:AB_88247, http://scicrunch.org/resolver/AB_88247.

[27] RRID:AB_2714189, http://scicrunch.org/resolver/AB_2714189.

[28] RRID:AB_330924, http://scicrunch.org/resolver/AB_330924.

[29] Bauriaud-Mallet M, Vija-Racaru L, Brillouet S, et al. The cholesterol-derived metabolite dendrogenin A functionally reprograms breast adenocarcinoma and undifferentiated thyroid cancer cells. J Steroid Biochem Mol Biol. 2019;192:105390. 31170473 10.1016/j.jsbmb.2019.105390

[30] Xia W, Li D, Wang G, et al. Small activating RNA upregulates NIS expression: promising potential for hepatocellular carcinoma endoradiotherapy. Cancer Gene Ther. 2016;23(10):333-340. 27608773 10.1038/cgt.2016.36

[31] Vitale M, Di Matola T, D’Ascoli F, et al. Iodide excess induces apoptosis in thyroid cells through a p53-independent mechanism involving oxidative stress. Endocrinology. 2000;141(2):598-605. 10650940 10.1210/endo.141.2.7291

[32] Golstein J, Dumont JE Cytotoxic effects of iodide on thyroid cells: difference between rat thyroid FRTL-5 cell and primary dog thyrocyte responsiveness. J Endocrinol Invest. 1996;19(2):119-126. 8778164 10.1007/BF03349847

[33] Pereira A, Braekman JC, Dumont JE, Boeynaems JM Identification of a major iodolipid from the horse thyroid gland as 2-iodohexadecanal. J Biol Chem. 1990;265(28):17018-17025. 2211608

[34] B’chir W, Maurin AC, Carraro V, et al. The eIF2α/ATF4 pathway is essential for stress-induced autophagy gene expression. Nucleic Acids Res. 2013;41(16):7683-7699. 23804767 10.1093/nar/gkt563 PMC3763548

[35] Son Y, Cheong YK, Kim NH, Chung HT, Kang DG, Pae HO Mitogen-activated protein kinases and reactive oxygen species: how can ROS activate MAPK pathways? J Signal Transduct. 2011;2011:792639. 21637379 10.1155/2011/792639 PMC3100083

[36] Serrano-Nascimento C, da Silva Teixeira S, Nicola JP, Nachbar RT, Masini-Repiso AM, Nunes MT The acute inhibitory effect of iodide excess on sodium/iodide symporter expression and activity involves the PI3K/Akt signaling pathway. Endocrinology. 2014;155(3):1145-1156. 24424051 10.1210/en.2013-1665

[37] Yao X, Li M, He J, et al. Effect of early acute high concentrations of iodide exposure on mitochondrial superoxide production in FRTL cells. Free Radic Biol Med. 2012;52(8):1343-1352. 22330063 10.1016/j.freeradbiomed.2012.02.002

[38] Zhang D, Xu X, Li J, et al. High iodine effects on the proliferation, apoptosis, and migration of papillary thyroid carcinoma cells as a result of autophagy induced by BRAF kinase. Biomed Pharmacother. 2019;120:109476. 31563816 10.1016/j.biopha.2019.109476

[39] van Staveren WC, Solís DW, Delys L, et al. Human thyroid tumor cell lines derived from different tumor types present a common dedifferentiated phenotype. Cancer Res. 2007;67(17):8113-8120. 17804723 10.1158/0008-5472.CAN-06-4026

[40] Landa I, Pozdeyev N, Korch C, et al. Comprehensive genetic characterization of human thyroid cancer cell lines: a validated panel for preclinical studies. Clin Cancer Res. 2019;25(10):3141-3151. 30737244 10.1158/1078-0432.CCR-18-2953 PMC6522280

